# Ovarian thecoma-fibroma groups: clinical and sonographic features with pathological comparison

**DOI:** 10.1186/s13048-016-0291-2

**Published:** 2016-11-22

**Authors:** Hui Chen, Yan Liu, Li-fei Shen, Mei-jiao Jiang, Zhi-fang Yang, Guo-ping Fang

**Affiliations:** 1 Department of Obstetrics and Gynecology, Ruijin Hospital, Shanghai Jiaotong University School of Medicine, Shanghai, 200025 People’s Republic of China; 2 Department of Ultrasound, Ruijin Hospital, Shanghai Jiaotong University School of Medicine, Shanghai, 200025 People’s Republic of China; 3 Department of Pathology, Ruijin Hospital, Shanghai Jiaotong University School of Medicine, Shanghai, 200025 People’s Republic of China

**Keywords:** Thecoma, Fibroma, Ovarian neoplasms, Ultrasound, Pathology

## Abstract

**Background:**

Ovarian thecoma-fibroma groups (OTFG) are uncommon sex cord-stromal neoplasms. The objective of the study was to demonstrate clinical and sonographic features of OTFG and compare with surgical histopathology.

**Methods:**

A total of 61 patients with surgically proven OTFG were enrolled in this retrospective study to demonstrate its clinical and sonographic features and to compare with pathological findings. Gray scale and color Doppler sonography were performed presurgically with either transabdominal or transvaginal approach to image pelvic structures and lesions. The clinical findings and sonographic appearances were compared with the types of the OTFG tumors based on the histopathological diagnosis.

**Results:**

The mean patient age was 53.57 (range, 26–86) years. There were 63.93% (39/61) patients in postmenopausal and 63.93% (39/61) patients with no clinical symptoms. Ultrasound findings of OTFG revealed as solid tumors with a typical feature of well-demarcated hypoechoic masses in 70.49% (43/61), among which 74.41% (32/43) tumors were smaller than 5 cm in diameter. There were 17 mixed echogenic masses with calcification, hemorrhage, or cyst, among which 70.59% (12/17) lesions were larger than 5 cm in diameter. Acoustic attenuation of the tumor was presented in 44.26% (27/61) of the cases. Doppler flow signals within the tumors were found in 20 cases (32.79%), in which 80% (16/20) had minimal or moderate flow signals. Ascites was detected in 32.79% (20/61) of the cases, Megi’s syndrome was found in 1 case. Final pathology revealed 41 (67.21%) thecoma-fibromas, 15 (24.59%) fibromas, 4 (6.56%) thecomas and 1 (1.64%) fibrosarcoma. There were 58 patients underwent cancer antigen 125 (CA125) test, and 20.69% (12/58) showed an elevated level. The diameter of tumors was found to be significantly correlated with CA125 level (*p* < 0.01) and the amount of ascites fluid (*p* < 0.05).

**Conclusions:**

The typical sonographic features of OTFG include adnexal hypoechoic masses with clear border and acoustic attenuation as well as minimal Doppler flow signals. All the aforementioned features could make ultrasound imaging as a assistent tool improve the preoperative diagnostic accuracy.

## Background

Ovarian thecoma-fibroma groups (OTFG) are uncommon sex cord-stromal neoplasms, constituting 1.0 to 4.0% of all ovarian tumors [1–4]. Most of them are benign, and often found in postmenopausal patients [5, 6]. The fibromas are composed in variable proportion of spindle cells forming collagen, while thecomas arise from stromal cells which resemble the perifollicular thecal cells, and occasionally there are histologic features of both fibroma and thecoma, giving rise to the term fibrothecoma. According to the World Health Organization classification of ovarian neoplasms, they belong to OTFG neoplasms [5, 7–9]. Plain Computerized Tomography (CT) scan shows hypodensity or isodensity of the tumors while contrast enhanced CT scan mainly shows no enhancement or delayed mild enhancement. Low signals of the tumors are shown by T1-weighted and T2-weighted Magnetic Resonance Imaging (MRI), slightly enhanced by MRI contrast agent [10–12]. However, the preoperative diagnostic rate of OTFG is rather low due to its low incidence, diverse clinical syndromes, and the great differences existing in tumor size, shape and internal components. It is therefore often misdiagnosed as uterine myoma. When the tumor size is large, with ascites or even hydrothorax, and an elevated cancer antigen 125 (CA125) level, it is probably misdiagnosed as malignancy [5]. Proper preoperative diagnosis of the disease, therefore, has significant implications for clinical treatment. Currently, there is little research investigating the relationship between ultrasound features and pathologic features of OTFG. The present study collected and analyzed the ultrasound features, as well as the clinical and pathologic findings of OTFG, with the aim to improve preoperative diagnostic accuracy.

## Methods

### Clinical data

In this retrospective study, 61 patients with surgically proven OTFG neoplasms were enrolled from Shanghai Ruijin Hospital between June 2009 and November 2015. The clinical information, CA125 test and sonographic results of the OTFG tumors were analyzed and compare to pathological findings. The study was approved by the Ruijin Hospital, Shanghai Jiaotong University School of Medicine institutional ethics committee with exemption to obtain informed consent from individual patients.

### Ultrasound examination

All 61 patients underwent pre-surgical ultrasound examination of the pelvis using iU22 and HD11 ultrasound machines (Philips Health Systems, Bothell, WA, USA) associated with a 7.0–9.0 MHz transvaginal and a 3.5 MHz transabdominal probes. Ultrasound parameters were adjusted to optimize the image quality for each patient. Multi-dimensional and multi-angle real-time scans were performed to image the uterus, adnex and masses of their locations, sizes, patterns, echogenic characteristics, color Doppler flow, pelvic effusion, and relationship with surrounding organs.

Color Doppler flow imaging within each tumor was recorded and Doppler signal was scored according to established standard by D. Timmerman et al. [13], i.e., Score 1: no color flow signals detected, Score 2: only minimal color signals detected, Score 3: moderate color signals displayed and Score 4: abundant color signals presented. Both grey scale and Doppler ultrasound images with typical features were digitally recorded in the hard drive of the system. The impression and preliminary diagnosis of the ovarian tumors based on the grey scale and Doppler follow imaging was made for each case.

### Pathological examination

The surgical pathological specimens were immediately fixated in 4% formaldehyde and embedded in paraffin. The sectioned slides were stained with hematoxylin-eosin (HE) for histopathological assessment using light microscopes.

Each patient of clinical information, CA125 test and ultrasound results were analyzed and compared to intraoperative findings and final pathological diagnosis.

### Statistical analysis

All of the statistical analyses were performed using SPSS 13.0 (SPSS Inc., Chicago, IL, USA). Measurement data were expressed as mean ± standard deviation (x ± s). *T*-test was used for comparing the means of two samples; *χ* ^2^ test was used for comparing measurement data; The Pearson’s Correlation was also performed to show the relationship among the diameter of tumors and the CA125 levels and the amount of ascites fluid. Statistical significance was assumed at a *p*-value < 0.05.

## Results

### Clinical information

The mean ± SD age of 61 patients was 53.57 (±15.52; range, 26–86) years. There were 65.57% (40/61) patients over 50 years old and 63.93% (39/61) in postmenopausal. Among the 61 patients, the neoplasms were discovered by routing physical exam in 39 (63.93%) cases and hospital visiting for abdominal pain or bulge in 16 (26.23%) cases, for menstrual disorders in 4 (6.56%) cases and for postmenopausal bleeding in 2 (3.28%) cases.

Among 58 of 61 patients who underwent CA125 test, 12 (20.69%) patients showed increase value with a mean of 166.67 U/ml ranged from 37.6 U/ml to 456.2U/ml (Normal Value: <35U/ml). In the 12 cases showing an elevated level of CA125, 91.67% (11/12) had a diameter of the tumor greater than 5 cm. By Pearson’s Correlation analysis, there was a statistically significant correlation between the diameter of tumors and CA125 level (*r* = 0.64, *p* < 0.01).

### Ultrasound findings

**Fig. 1:**
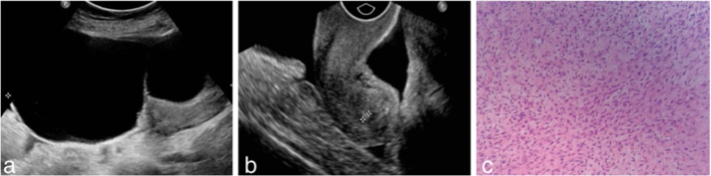
A 63-year-old woman with pelvic mass was found by physical examination. **a** A 133 mm*69 mm*169 mm well-circumscribed mixed mass was detected in the right ovary by ultrasound examination; **b** ultrasound detected ascites in the pelvis; **c** pathological findings showed ovarian thecofibroma with collagen change

**Fig. 2:**
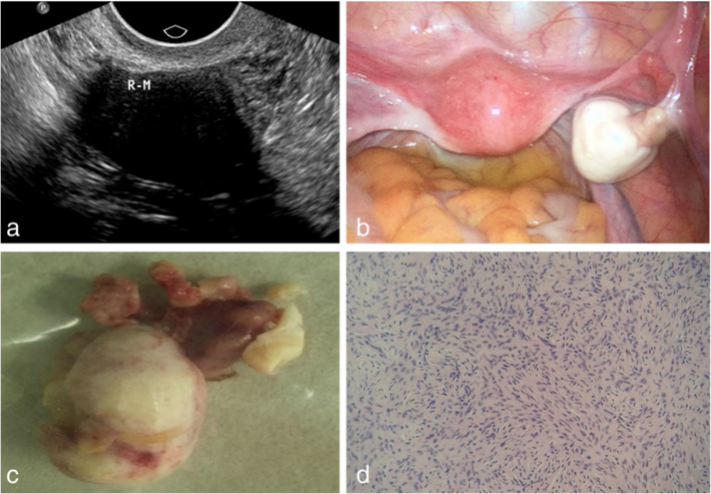
A 53-year-old woman with a pelvic mass discovered by routing physical examination. **a** On ultrasound examination, a 28 mm*22 mm*26 mm well-circumscribed hypoechoic mass was observed in the right ovary, with posterior echo attenuation. **b** On laparoscopy surgery, right ovarian was hard and enlarged, the surface smooth, having a good mobility; in the pelvic, a small number of pale yellow ascites were observed. **c** Pathologically, right ovarian was enlarged, with tough textures and grayish white surfaces. **d** Pathological findings confirmed thecofibroma in right ovarian

**Fig. 3:**
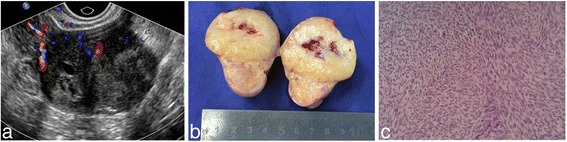
A 53-year-old pre-menopausal woman having a pelvic mass was detected by physical examination. **a** On ultrasound examination, a 28 mm*22 mm*26 mm well-demarcated hypoechoic mass with small anechoic areas was observed at left ovary, and moderate Doppler signals were seen in the tumor; **b** the tumor section showed grey-white appearance and yellowish areas with hemorrhage in the center of the tumor; **c** pathological findings proved to be cellular fibroma with partial growth activity

Sonographic findings of the neoplasms in 61 patients were presented in Table 1 and Figs. 1 (a, b), 2 (a) and 3 (a). The typical sonographic feature of the OTFG was well-demarcated hypoechoic mass with smooth and clear margin in 70.49% (43/61). The mean diameter of the tumor was 5.86 cm, ranging from 1 ~ 25 cm, Based on preoperative ultrasound features, specific diagnosis of OTFG were correctly made in 72.13% (44/61) cases while 12 (19.67%) cases were misdiagnosed as subserous myoma, 1 (1.64%) as ovarian malignancy, 2 (3.28%) as endometrioma, 1 (1.64%) as complex cyst and 1 (1.64%) as cystadenomas.

**Table 1:** Ultrasound features in 61cases with OTFG neoplasms

	Number	Percentage (%)
Diameter (cm)		
< 5	37	60.66
≥ 5,<10	15	24.59
≥ 10	9	14.75
Echogenicity of the tumor		
Hypoechoic	43	70.49
Mixed echoic	17	27.87
Anechoic	1	1.64
Echo attenuation	27	44.26
Doppler flow signal		
Score 1: (None)	41	67.21
Score 2: (Minimal)	14	22.95
Score 3: (Moderate)	2	3.28
Score 4: (Abundant)	4	6.56

### Surgery and pathological results

Intraoperative findings revealed 65 tumors in 61 patients, in which 95.08% (58/61) of the lesions occurred unilaterally. There were one patient with two lesions located in the same side and 3 patients within 6 lesions found bilaterally. In 61 cases, 4/65 (6.15%) tumors were not identified by ultrasound examination before the surgery. Ascites was found in 20 cases (32.79%) with the amount of fluid ranging from 50 ml to 800 ml while 1 case showed Megi’s syndrome, i.e., with massive abdominal and pleural effusion. In the 20 cases with ascites, 80% (16/20) of the tumors had a diameter of the tumor greater than 5 cm. Pearson’s analysis showed a statistically significant correlation between the diameter of tumors and the amount of ascites fluid (*r* = 0.50, *p* < 0.05). The gross appearances of OTFG neoplasms were described as round, oval, lobulated or well-circumscribed solid tumors. The section of the tumor appeared as grayish white or yellow with edema, cystic degeneration, or necrosis. Microscopically, the tumor cells were spindle or short spindle with feather-like or interwoven arrangements. Nucleus of the cell was round or oval, accompanied by calcification, collagen change, and hyalinization degeneration in the tumors. Final pathologic diagnosis had 42 thecofibromas (including 3 cellular thecofibromas), 16 fibromas (including 4 cellular fibromas), 5 thecomas (including 1cellular thecomas), 1 fibrothecoma and 1 fibrosarcoma. Pathological findings are presented in Figs. 1 (c), 2 (b, c, d) and 3 (b, c). The Comparison of ultrasound features and pathological findings were presented in Table 2.

**Table 2:** Ultrasound features and pathological findings of the 61 OTFG tumors

Ultrasound features	Thecofibroma (n)	Fibroma (n)	Thecoma (n)	Fibrosarcoma (n)	Total (n)
Hypoechoic	32	7	3	1	43
Mixed echoic	8	8	1	0	17
Anechoic	1	0	0	0	1
Total	41	15	4	1	61

After surgery, 55 (90.16%) patients were followed up for an average of 10.6 months from 3 to 72 months. Postsurgical recurrence was not observed in any cases (including one patient with fibrosarcoma) by clinical and sonographic examination. In one patient with Megi’s syndrome, ascites and pleural effusion disappeared three months after operation. In addition, two patients had full term deliveries 1 year after tumor removal.

## Discussion

OTFG neoplasms mostly occur in postmenopausal women, often with a good prognosis. In this study, 63.93% (39/61) of the lesions occurred after menopause, with 65.57% (40/61) in women aged 51 to 70. Most patients visited the hospital because of pelvic masses found in routing physical examination, while 63.93% (39/61) of these patients showed no obvious clinical symptoms [5]. Symptoms such as abdominal pain and abdominal bulge may occur in cases with large tumors. Among the 16 patients who visited the hospital for abdominal pain or bulge, 81.25% (13/16) had a tumor with a diameter over 5 cm. Thecoma is benign lesion and occasionally malignant, which may secrete estrogen and lead to symptoms such as menstrual disorders and postmenopausal bleeding [14]. Among the sample population,fibromas and thecofibromas account for 91.80% (56/61) of all tumors which usually show no postmenopausal bleeding or menstrual disorders.

The most tumors were unilateral, and few were bilateral [11]. In our study, surgery revealed that 91.80% (58/61) of the lesions occurred unilaterally, and 3 cases occurred bilaterally. In 20 cases (32.79%),minimal and moderate ascites was present in this study which consistent with literature reports [15]. Megi’s syndrome occurred in one case (1/61, 1.64%) in our serial with similar incident rate by other study [3]. Correlation analysis revealed that the diameter of tumors was statistically significantly correlated with CA125 level and the amount of ascites fluid (*p* < 0.05) which consistent with previous report [16]. Ascites formation may be due to transudation through the tumor surface which exceeds the peritoneum’s resorptive capacity [17]. Irritation of the peritoneal surface by the tumor may explain the increased CA 125 levels [5].

Pathologically, OTFG neoplasms are tumors of sex-cord stromal origin and the most of them contain both theca cells and fibroblasts. Of all tumors identified in this study, 41/61 (67.21%) tumors were histologically proved to be thecofibroma. Comparison of ultrasound and pathologic findings of OTFG indicated that typical sonographic features of OTFG were adnexal hypoechoic masses with clear border and acoustic attenuation,which consistent with previous findings [11, 15]. The sonographic features varied in accordance with tumor components and degeneration, or complicated with other cystic lesions [8]. In 4 patients with thecoma (histologically composed theca cells), ultrasound imaging showed uniform hypoechogenic appearance with attenuation and relatively small in size (less than 5 cm in diameter), similar to what was found by Eike Burandt et al. [14]. Pathologically, thecofibroma and fibroma were described to be more component of fibroblast and less of theca cells. Therefore, these two types of tumors showed no dramatic differences on ultrasound imaging, usually described as adnexal hypoechoic masses. However, among tumors with a diameter larger than 5 cm, 95.83% (23/24) of the tumor were diagnosed as thecofibroma or fibroma; while among tumors with a diameter larger than 10 cm, 77.79% (7/9) of the tumor were thecofibroma, with some difference found by Chung BM et al. [18].

Large tumors were often associated with torsion, hemorrhage, calcification, or complicated with other cystic lesions, which showed mixed echogenic masses. In 17 tumors (27.87%) with mixed echogenic appearance, there were 8 thecofibroma, 8 fibroma and 1thecoma in which all were associated with other complicated lesions including endometrioma (*n* = 3), teratoma (*n* = 1), cystadenomas (*n* = 2), 1 case with hemorrhage (*n* = 1), ovary torsion (*n* = 3), calcification or degeneration changes (*n* = 7).

Among the 27 tumors that showed acoustic attenuation, 96.30% (26/27) were thecofibroma and fibroma. The reason of the attenuation is most likely due to the low sound propagation of fibroblast tissue [19]. In general, the blood flow of OTFG tumor is not rich. Minimal or moderate Doppler flow signals were detected in 26.23% (16/61) tumorsn while 4 (6.56%) tumors showed abundant flow signals. Therefore, accurate diagnoses can be made based on sonographic characterizations such as adrenal hypoechoic masses with clear border, posterior echo attenuation and minimal or moderate blood flow signals inside the tumors. In the presence of adnexal mixed echoic masses, tumor degeneration and with other tumor should be considers. On the whole, sonographic findings can reflect pathological changes.

In this study, ultrasound examination made correct diagnosis of OTFG in 44 (72.12%, 44/61) tumors before surgery. Four tumors less than 2 cm in diameter failed to be detected by ultrasound, among which 3 cases had bilateral lesions and 1 case had 2 lesions on the same side. The detection rate of tumors was 93.85% (61/65). Failure to detect some tumors may be caused by small size of the tumors as well as leaving out presence of contralateral lesions.

In early days with limited understanding of OTFG tumors, it was often confused with subserous myoma on ultrasound imaging [6]. In the present study, 12 tumors were misdiagnosed as subserous myoma in patient with a mean age of 53.57 years and 63.93% postmenopausal with small ovaries. Based on our experiences, several techniques can be considered to differentiate OTFG neoplasms from myoma: 1. uterine myoma often happened in women of reproductive age instead of postmenopausal women, 2. the myoma’s capsule of connected with the uterine serosa and with blood supply from uterine branches, and 3. the masses would be separated from the uterus with use of manipulation of abdominal compression. Therefore, a solid mass detected next to the uterus in postmenopausal woman should not only considerate as a subserous myoma, in instead of, the OTFG neoplasm should be ruled out, especially when ipsilateral ovarian is not displayed.

With technological development, high resolution ultrasonography and contrast-enhanced ultrasound imaging have become one of the important imaging modality for clinical diagnosis and differentiation of uterine and ovarian neoplasms [20]. A literature review shows that ovarian fibroma and subserous myoma have clear differences in contrast-enhanced ultrasound imaging. The fibroma showed hypoenhancement with a delay perfusion of the tumor compared to the uterus while the subserous myoma exhibited homogeneous isoenhancement and synchronous enhancement with the myometrium in early phase [21].

In this study, one cellular thecofibroma was misdiagnosed as malignancy because it was irregular multilocular solid tumor with abundant blood flow, which was a sign of malignancy. Two other misdiagnosed thecofibroma revealed a complex ovarian cyst with homogeneous internal echoes, which contained a small solid-appearing area with undetectable flow in it. These two thecofibroma were misdiagnosed as endometriomas. These misdiagnosis based on ultrasound findings were consistent with previous studies [7, 22, 23].

There are several limitations of the study. First, this is retrospective research that may lack control and desirable study design. Second, all patient’s data is reviewed and analyzed without blind fashion. Third, the study only focused on the OTFG neoplasms and did not include other ovarian tumors for comparison. Further study with prospective design and comparison with other ovarian tumors is needed to provide additional information for differential diagnoses of ovarian tumors.

## Conclusion

OTFG neoplasms often occur in postmenopausal women with no obvious clinical symptoms. Most of them are benign, with a good prognosis. The typical sonographic features of OTFG are adnexal hypoechoic masses with clear border and acoustic attenuation as well as minimal Doppler flow signals. They are often unilateral with a diameter smaller than 5 cm. Combined with clinical information and CA125, ultrasound imaging could be used as an imaging tool for improving the preoperative diagnostic accuracy.

## Abbreviations

CA125: Cancer antigen 125
CT: Computerized tomography
HE: Hematoxylin-eosin
MRI: Magnetic resonance imaging
OTFG: Ovarian thecoma-fibroma groups
